# Climatic Signals from Intra-annual Density Fluctuation Frequency in Mediterranean Pines at a Regional Scale

**DOI:** 10.3389/fpls.2016.00579

**Published:** 2016-05-02

**Authors:** Enrica Zalloni, Martin de Luis, Filipe Campelo, Klemen Novak, Veronica De Micco, Alfredo Di Filippo, Joana Vieira, Cristina Nabais, Vicente Rozas, Giovanna Battipaglia

**Affiliations:** ^1^Department of Agricultural Sciences, University of Naples Federico IIPortici, Italy; ^2^Department of Geography and Regional Planning, Environmental Sciences Institute, University of ZaragozaZaragoza, Spain; ^3^Department of Life Sciences, Centre for Functional Ecology, University of CoimbraCoimbra, Portugal; ^4^Department of Agricultural and Forestry Science, Tuscia UniversityViterbo, Italy; ^5^Departamento de Ciencias Agroforestales, Escuela Universitaria de Ingenierías Agrarias, Universidad de Valladolid, Campus Duques de SoriaSoria, Spain; ^6^Department of Environmental, Biological and Pharmaceutical Sciences and Technologies, Second University of NaplesCaserta, Italy; ^7^Laboratoire Paléoenvironnements et Chronoécologie, Ecole Pratique des Hautes Etudes, Institut des Sciences de l’Evolution – UMR 5554, Université de MontpellierMontpellier, France

**Keywords:** IADF, tree rings, climate, *Pinus halepensis*, *Pinus pinea*, *Pinus pinaster*

## Abstract

Tree rings provide information about the climatic conditions during the growing season by recording them in different anatomical features, such as intra-annual density fluctuations (IADFs). IADFs are intra-annual changes of wood density appearing as latewood-like cells within earlywood, or earlywood-like cells within latewood. The occurrence of IADFs is dependent on the age and size of the tree, and it is triggered by climatic drivers. The variations of IADF frequency of different species and their dependence on climate across a wide geographical range have still to be explored. The objective of this study is to investigate the effect of age, tree-ring width and climate on IADF formation and frequency at a regional scale across the Mediterranean Basin in *Pinus halepensis* Mill., *Pinus pinaster* Ait., and *Pinus pinea* L. The analyzed tree-ring network was composed of *P. pinea* trees growing at 10 sites (2 in Italy, 4 in Spain, and 4 in Portugal), *P. pinaster* from 19 sites (2 in Italy, 13 in Spain, and 4 in Portugal), and *P. halepensis* from 38 sites in Spain. The correlations between IADF frequency and monthly minimum, mean and maximum temperatures, as well as between IADF frequency and total precipitation, were analyzed. A significant negative relationship between IADF frequency and tree-ring age was found for the three Mediterranean pines. Moreover, IADFs were more frequent in wider rings than in narrower ones, although the widest rings showed a reduced IADF frequency. Wet conditions during late summer/early autumn triggered the formation of IADFs in the three species. Our results suggest the existence of a common climatic driver for the formation of IADFs in Mediterranean pines, highlighting the potential use of IADF frequency as a proxy for climate reconstructions with geographical resolution.

## Introduction

Tree-ring width is a powerful proxy of past environmental conditions able to record fluctuations of biotic and abiotic factors during the tree’s lifetime (Fritts, 2001). Tree rings reveal physiological response to environmental fluctuations because the latter affect xylogenesis which in turn can lead to peculiar anatomical features, such as intra-annual density fluctuations (IADFs). IADFs are defined as a layer of cells within a tree ring identified by different shape, size, and wall thickness (Kaennel and Schweingruber, 1995), and characterized by the occurrence of latewood-like cells within earlywood or earlywood-like cells within latewood (Fritts, 2001). They can occur in several species in different environments and are often irregularly found in time and space (Cherubini et al., 2003; De Micco et al., 2016). IADFs constitute a useful tool to reconstruct intra-annual changes in climatic factors, providing detailed information at the seasonal level (Rigling et al., 2001, 2002; Copenheaver et al., 2006; Campelo et al., 2007, 2013; de Luis et al., 2007, 2011a; Bogino and Bravo, 2009; Hoffer and Tardif, 2009; Battipaglia et al., 2010; Edmonson, 2010; Vieira et al., 2010; Rozas et al., 2011; Olivar et al., 2012; Novak et al., 2013a,b; Nabais et al., 2014; Olano et al., 2015). IADF formation can be considered as a strategy of trees to adjust wood anatomical traits to short-term variations in environmental conditions maintaining the balance between hydraulic efficiency and safety against embolism during wet and dry periods, respectively (Campelo et al., 2007; De Micco et al., 2007; De Micco and Aronne, 2009; Wilkinson et al., 2015). Numerous studies reported high IADF frequency in species growing in the Mediterranean area, which is considered one of the most vulnerable regions to climate changes. According to the Intergovernmental Panel on Climate Change [IPCC] (2014), higher irregularities in the intra-annual precipitation patterns and increasing temperature are expected in the Mediterranean Basin in the next decades (Giorgi and Lionello, 2008). The expected climate changes will likely have an impact on tree growth and thus IADF frequency.

Most dendrochronological studies on IADF occurrence in the Mediterranean area have been conducted on *Pinus* species, since Mediterranean pines are quite sensitive to climate fluctuations and are prone to form IADFs (Campelo et al., 2007, 2013, 2015; de Luis et al., 2007, 2011a; Carvalho et al., 2015; De Micco et al., 2007; Vieira et al., 2009, 2010, 2015; Rozas et al., 2011; Olivar et al., 2012; Novak et al., 2013a,b; Nabais et al., 2014; Carvalho et al., 2015). Despite the variety of climatic conditions throughout the Mediterranean Basin, *Pinus* is a widespread genus (Barbéro et al., 1998), allowing to compare the climate response of different species at a regional scale.

IADF formation is reported to depend on tree age, sex, size, and/or width of the formed tree-ring (Rigling et al., 2001; Wimmer, 2002; Campelo et al., 2007, 2013, 2015; Bogino and Bravo, 2009; de Luis et al., 2009; Vieira et al., 2009; Olivar et al., 2012; Nabais et al., 2014; Olano et al., 2015). As a consequence, a wide variability in the occurrence of IADFs across species distribution is commonly described (Rigling et al., 2002; Edmonson, 2010; Novak et al., 2013b; Nabais et al., 2014). A higher frequency of IADFs has been found in young trees of *Pinus pinaster* growing under Mediterranean climate compared to older ones (Bogino and Bravo, 2009; Vieira et al., 2009). A similar age-relation has been observed in *Pinus halepensis* stands throughout its natural distribution area in the Iberian Peninsula (Olivar et al., 2012; Novak et al., 2013b). In the Iberian Peninsula, an age and size dependency of IADF frequency in *P. halepensis* and *P. pinaster* trees has been reported: the maximum frequency of IADFs was observed during the juvenile stages (about 27 years-old trees), and more IADFs were found in wider than narrower tree rings (Novak et al., 2013b; Campelo et al., 2015). In *P. pinaster* from east-central Spain, the presence of IADFs has been negatively correlated with radial growth rates (Bogino and Bravo, 2009), while no significant relationships of IADF frequency with age and tree-ring width have been found in young trees (<55 years) from the wetter north-western Spain (Rozas et al., 2011). Aside from Mediterranean pines, significant relationships between IADF frequency and either tree-ring age (negative) or tree-ring width (positive) have been found in *Pinus sylvestris* trees growing in dry sites in the central Alps (Rigling et al., 2001, 2002). Analyzing tree rings of *Pinus banksiana* and *Picea mariana* from eastern Manitoba, Hoffer and Tardif (2009) showed a higher frequency of IADFs in juvenile rings than in older ones, but no significant relation between IADF occurrence and tree-ring width was found.

A few studies have been performed on the geographical variation of IADF occurrence. A significant variability in the frequency of IADFs across the range of *P. halepensis* was found in Spain (Novak et al., 2013b), with a higher frequency of IADFs in coastal sites than inland or mountain sites. Moreover, Rozas et al. (2011) found that IADF frequency of *P. pinaster* under Atlantic climate depends strongly on elevation, with more abundant IADFs at low elevations. Rigling et al. (2002) showed a higher mean IADF frequency in *P. sylvestris* growing at a drier than moderate wet sites in Switzerland. A recent study on *P. pinaster* and *Pinus pinea* comparing a Mediterranean and a temperate site in Portugal highlighted that local adaptation and site-specific climatic conditions can play an important role in the formation of IADFs regardless of the species (Nabais et al., 2014).

The literature survey reveals that available data about the relations between IADFs and climate were based on single species or when more than one species was used they were restricted to a single or a few sites. Indeed, studies based on a network of IADFs covering a broad geographical area would likely help to gain information on the ability of tree species to adjust their hydraulic architecture and physiology in response to intra-annual environmental changes on a larger geographical scale.

In the present study, we used a network of IADF frequency covering a broad geographical area with the aim to analyze whether the occurrence of IADFs in Mediterranean pine species is triggered by common regional climatic drivers. In order to reach this aim, we investigated the relationships between IADF frequency and tree-ring age, tree-ring width and climate in three widespread Mediterranean pine species, namely *P. halepensis*, *P. pinaster* and *P. pinea*, growing along their distribution ranges.

Our specific goals were: (1) to characterize the regional patterns of IADF frequency in *P. halepensis*, *P. pinaster*, and *P. pinea* growing along their distribution range, (2) to determine *if* and *how* the relationships between IADF frequency and tree-ring age/width vary between the three species, and (3) to identify the large-scale climatic factors driving the formation of IADFs under Mediterranean climate, by analyzing the relationships between IADF frequency and monthly maximum temperature (T_max_), mean (T_mean_) and minimum temperature (T_min_), as well as total precipitation.

## Materials and Methods

### The Dataset: Species and Sites

The database consists of 55 previously published and 13 newly processed chronologies of tree-ring width and series of IADF frequency from: (a) *P. pinea* trees growing at 10 sites (i.e., 2 in Italy, 4 in Spain, and 4 in Portugal), (b) *P. pinaster* trees from 19 sites (i.e., 2 in Italy, 13 in Spain, and 4 in Portugal), and (c) *P. halepensis* trees from 38 sites in Spain. Details of each site are reported in the supporting material (Supplementary Table S1).

**FIGURE 1:**
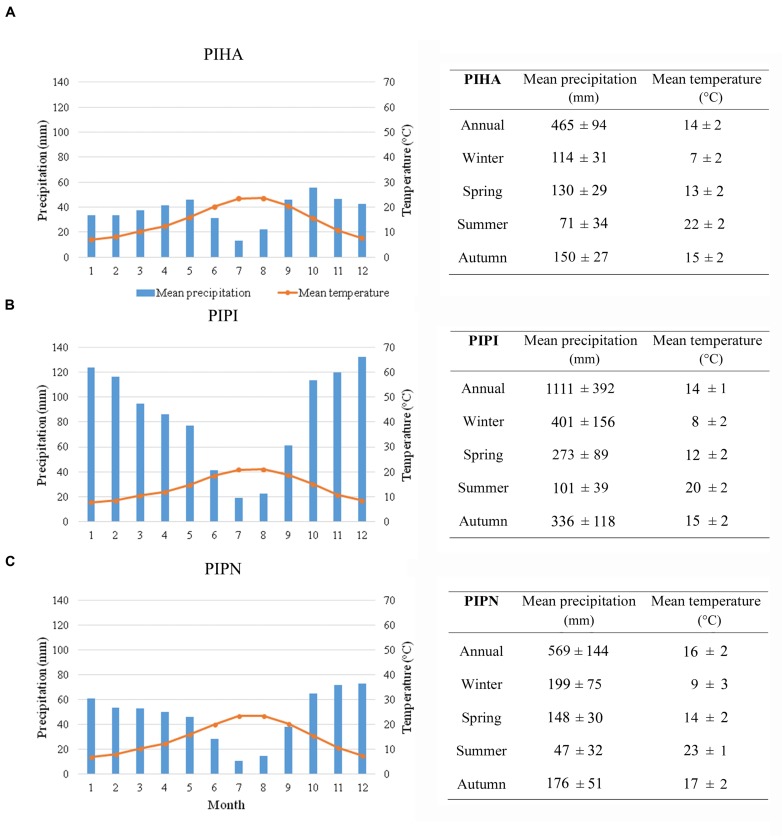
**Climate diagrams and seasonal and annual values of mean precipitation and mean temperature of the sites of the network grouped by species, for the period 1901–2013 [(A) ***Pinus halepensis*** (PIHA), (B) ***Pinus pinaster*** (PIPI), and (C) ***Pinus pinea*** (PIPN)].** Data from the CRU TS v. 3.22 dataset with 0.5° grid resolution (Harris et al., 2014).

Climatic time series of monthly temperature and total precipitation for the period 1901–2013, for all the sites, were derived from the Climatic Research Unit (CRU) TS v. 3.22 dataset with 0.5° grid resolution (Harris et al., 2014). CRU gridded data were chosen for comparative purposes because of its complete coverage of all studied sites and high correlations with the local weather stations. Mean monthly temperature and total precipitation as average of all the sites for each species are shown in climate diagrams in **Figure 1**. The overall climate regime is Mediterranean-like, with the occurrence of mild winter and spring, and a period of summer drought followed by an increase in precipitation concomitant to a decrease of temperature during autumn. The mean monthly temperature for all the study sites ranged from 7°C in January to about 24°C in August. The sites with the highest amount of precipitation were those where *P. pinaster* is dominant (**Figure 1B**): at these sites, the highest monthly values of precipitation during the entire year were recorded with a maximum in December (132.3 mm) and a minimum in July (18.8 mm). The lowest values of mean precipitation throughout the year were recorded for *P. halepensis* sites with a maximum of 55.6 mm in October and a minimum of 13.3 mm in July (**Figure 1A**). Finally, *P. pinea* trees grow in sites with the lowest amount of summer precipitation (**Figure 1C**) with July as the driest month (10.8 mm), and a maximum of rainfall in December (73.2 mm).

### Identification of IADFs

The database includes three species (*P. halepensis*, *P. pinaster*, and *P. pinea*) from a wide variety of sites. Despite the great potential of IADFs as proxies, the methods for their objective classification in different types (e.g., based on their relative position within the tree ring) have not been standardized yet. At present, to study IADFs, tree-ring series are mainly analyzed visually. Although, tree-ring width measuring and IADF identification were performed by different operators, all followed a common protocol and all were trained to adopt the same criteria for IADF identification. This allowed unambiguous identification of the presence/absence of IADFs, but the classification of different types of IADFs still suffered from subjectivity. Consequently, to build series of IADF occurrence, we adopted a conservative criterion using only the presence/absence of IADFs in tree rings visually identified on dated cores with the help of a stereomicroscope.

### Relationships between IADF Frequency and Tree-Ring Age and Width

The age of individual tree rings (here, defined as “tree-ring age”) was indicated in ascending order starting from the most juvenile ring to the oldest one within each core. To study the influence of tree-ring age on the likelihood of IADF formation, a logistic binomial model was applied using tree-ring age as the independent variable and the presence (1) or absence (0) of IADFs in the corresponding tree ring as the dependent variable. The analysis was conducted independently for each species and was limited to tree-ring ages with at least 20 tree rings. Data from a total of 84,794 tree rings ranging from tree-ring age 1 to 169 were included in the analysis for *P. halepensis.* The total number of tree rings analyzed for *P. pinaster* and *P. pinea* was 30,792 and 16,028, while the range of tree-ring age was from 1 to 125 years and from 1 to 108, respectively. Predicted values of IADF frequency obtained for each age class were used as reference series for detrending purposes.

The influence of tree-ring width on the likelihood of IADF formation was analyzed with a similar procedure and the same dataset by using a new set of logistic binomial models with the width of each individual tree ring as the independent variable. Predicted and detrended IADF values for each individual tree-ring width were calculated with the same method previously described for tree-ring age.

### IADFs Frequencies and Geographical Pattern

The geographical pattern of IADF frequency was analyzed using age-detrended IADF values obtained from the logistic binomial models including tree-ring data from all three species. To obtain detrended IADF values, the ratio between observed (0 or 1) and predicted IADF frequencies ([0,1]) was calculated for each individual IADF observation. Then, to obtain a robust estimation of the frequency of IADFs, independent from the age structures of the studied populations, the average of all individually obtained ratios (thereafter referred as IADF_r) were calculated for each study site. For each species, IADF_r equal to 1 represents years in which the frequency of IADFs is equal to the expected species-specific average. IADF_r of 2 and of 0.5 indicates that IADF frequency was twice and half the expected average, respectively. IADF_r were then rescaled to allow intra- and inter-species comparison. To do that, IADF_r obtained for each population was multiplied by the average IADF as predicted from age 1 to age 100 of the specific logistic model. The obtained rescaled frequency (IADF_f) represents the estimated frequency of IADFs for each population which is independent from the population age structure and comparable between sites and species.

### Replication Depth and a New Approach to Study Climatic Signal in IADFs

The principle of replication represents one of the keys of dendrochronological research highlighting the need to use more than one stem radius per tree and more than one tree per site to obtain reliable tree-ring chronologies. Different statistics based on mean inter-correlation among tree-ring series, as the expressed population signal (EPS), which determines how well a chronology established on a finite number of trees approximates the theoretical population chronology (Wigley et al., 1984; Briffa and Jones, 1990), are often used to identify well-replicated periods for different types of dendrochronological series (e.g., width, density, or chemical composition). Sampling strategies in dendrochronology are often designed to ensure such replication requirements.

However, the presence of other anatomical features like IADFs cannot be measured but just characterized as a binary variable of 0 and 1 (dummy variable), based on its absence or presence in a specific tree ring. In these cases, criteria to define the appropriate number of samples to obtain an accurate representativeness of IADF frequency cannot be based on the same approach used for tree-ring chronologies, due to the binary nature of the data. To determine the appropriate sample size needed to estimate the proportion of a population that possesses a particular property (i.e., IADF occurrence), a specific calculation needed to be computed (Eq. 1).

This equation allowed calculating the required sample size in order to estimate a proportion (prevalence) with a specified level of confidence and precision. For example, the number of required samples to estimate IADF frequency for a given site and a specific year, with a 95% of confidential level (*z* = 1.96) and a precision of 10% (*e* = 0.1), is 97 (Eq. 1).

(1) $$\begin{array}{r} n=(z^{2}*p\left(1-p\right))/e^{2}={\left[1.96\right]}^{2}\\ {}*0.5(1-0.5)/{\left[0.1\right]}^{2}=97 \end{array}$$

Indeed, this number is substantially higher than the number of samples that are commonly collected in dendrochronological research (based usually on 15 trees and 2 samples per tree). Thus, replication depth issue represents an important challenge aimed to obtain reliable estimations of the frequency of anatomical variables such as IADFs, especially when the aim is to identify the main climate factors promoting their formation. A well-defined sampling strategy could be the perfect solution to reach this purpose.

Nevertheless, to deal with this challenge we adopted an alternative analytical approach which allowed us to use data sets already available (previously collected for other dendro chronological purposes), but solving the problem associated to the high replication depth required.

Our approach was based on a global analysis by combining information from all the study sites and years. To study the influence of annual precipitation on IADF formation in a given species, all available individual tree rings were grouped in 100 classes according to the percentile positions of the local annual precipitation of the year of their formation. Tree rings were grouped in classes ranging from the ones formed under drier to those formed under wetter conditions. Then, mean annual precipitation and mean standardized IADF frequencies were calculated for each class. The statistical normality of the obtained IADF series was verified using the Kolmogorov–Smirnov’s test, then Pearson’s correlation coefficient was computed to study the association between the two series. By using such procedure, IADF frequencies were not calculated independently for any specific calendar year but estimated for different ranges of annual precipitation conditions. The estimation of frequency associated to each precipitation class was based on at least 158 samples (as for *P. pinea*), in agreement with replication requirements, since dataset including IADF quantification and climate data (1901–2013) included 79901, 30736, and 15889 tree rings for *P. halepensis*, *P. pinaster*, and *P. pinea*, respectively.

Furthermore, since IADF frequencies were not calculated on time series of tree rings in chronological order, but by grouping rings in classes according to the climate conditions occurring during their formation, autocorrelation did not affect the significance level of the results.

The same procedure as explained for annual precipitation was also applied to mean annual temperature (T_mean_), minimum (T_min_) and maximum temperature (T_max_) and total precipitation at monthly and seasonal scales from September of previous year to December of the current year. The correlations with temperature and precipitation of previous autumn months were performed to investigate the effect of growth conditions of the previous year on IADF frequency. The months of the whole calendar year were chosen for correlations between IADF frequency and current growth conditions, since cambial activity under Mediterranean climate was found to be active up to December (de Luis et al., 2009, 2011a,b).

## Results

Descriptive statistics and a summary for the measured variables from the three species are shown in **Table 1**. A total of 139,342 rings were analyzed for the three species considered together, of which 24,143 showed IADFs. Mean age varied among species and ranged between 38 and 48 years. Mean tree-ring width ranged between 1.77 mm for *P. halepensis* and 2.76 mm for *P. pinaster*.

**Table 1:** Descriptive statistics and measured variables for the three species (PIHA, *Pinus halepensis*; PIPI, *Pinus pinaster*; PIPN, *Pinus pinea*).

	N of rings analyzed	Mean age (years)	Mean tree-ring width (mm)	N of rings with IADFs (raw frequency of IADFs)
PIHA	88375	48	1.77	7333 (0.08)
PIPI	33851	38	2.76	13161 (0.39)
PIPN	17116	45	2.36	3649 (0.21)

### IADF Frequency and Tree-Ring Age

**FIGURE 2:**
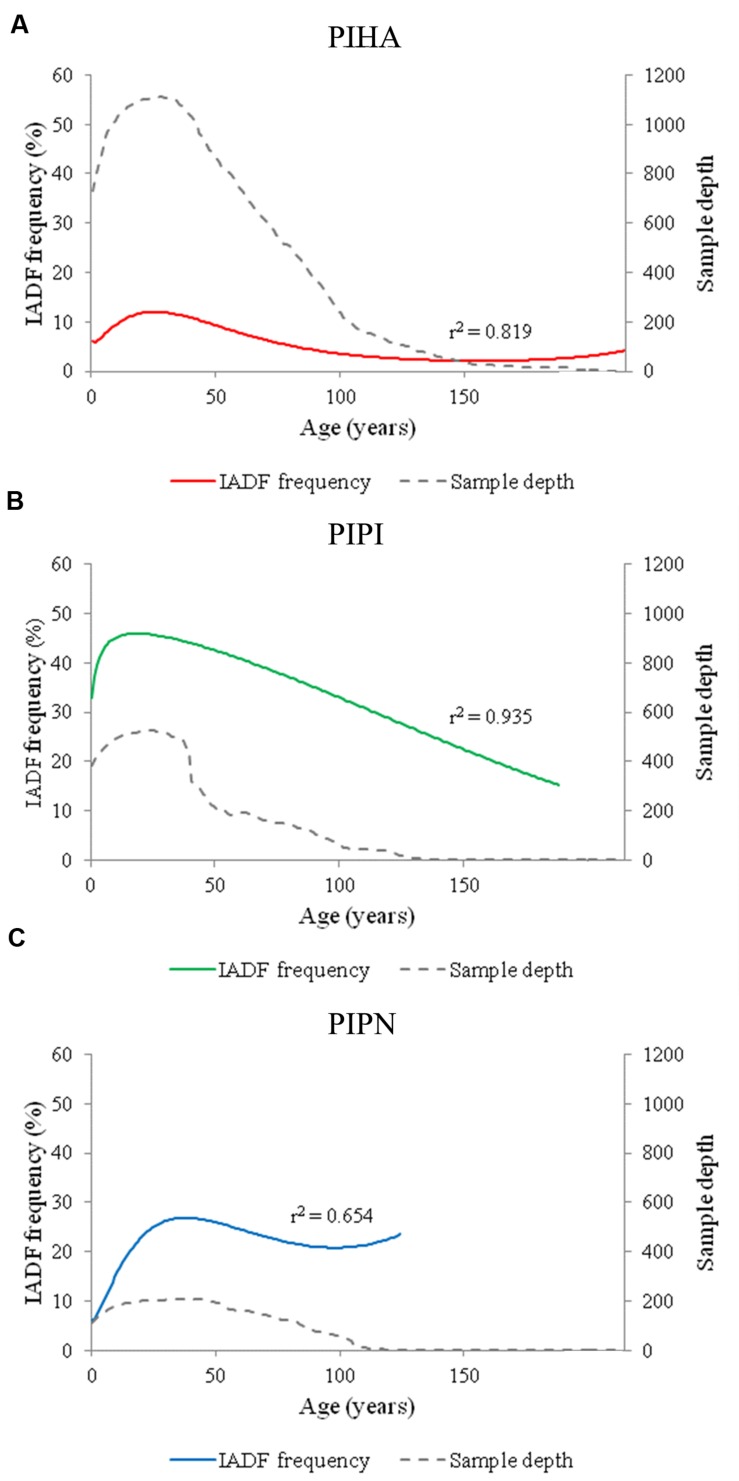
**Logistic binomial regressions between IADF frequency and tree-ring age for the three species (PIHA, ***Pinus halepensis***; PIPI, ***Pinus pinaster***; PIPN, ***Pinus pinea***).** Population structure and IADF frequency for **(A)** *Pinus halepensis* (PIHA), **(B)** *Pinus pinaster* (PIPI), and **(C)** *Pinus pinea* (PIPN). Age refers to tree-rings aligned from the most juvenile to the oldest ones.

An age-dependent trend was found in the distribution of IADF frequency for all the analyzed species (**Figures 2A–C**). Higher frequency of IADFs was found in juvenile than older rings in the three Mediterranean pines, with the peak shifting to different ring ages depending on the species. The logistic binomial regression between tree-ring age and the IADF frequency showed an asymmetric bell-shaped distribution with a maximum of 12% at the age of 26 years in *P. halepensis*, of 45.9% at the age of 19 years in *P. pinaster* and of 26.9% at the age of 38 years in *P. pinea*. Sample depth per each species is shown in **Figure 2**.

### IADF Frequency and Tree-Ring Width

**FIGURE 3:**
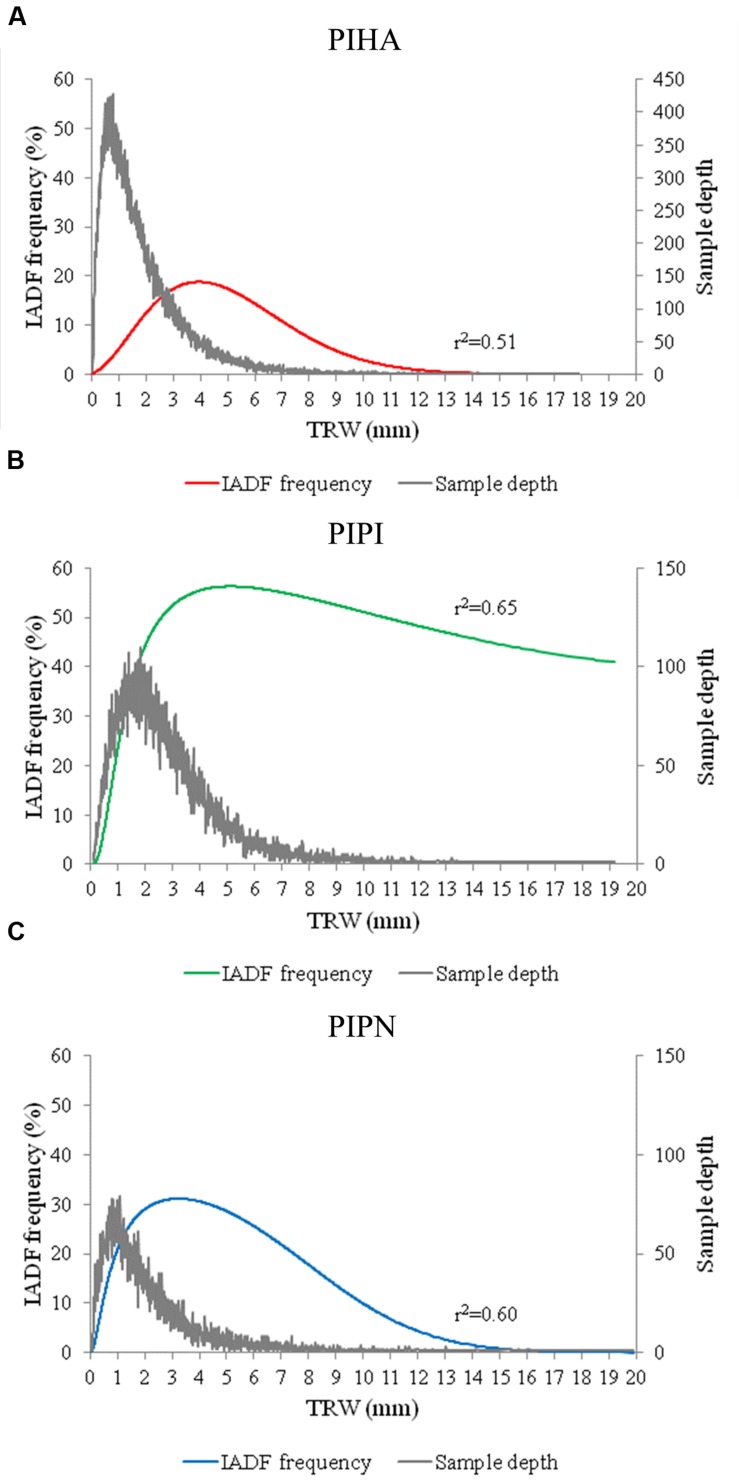
**Logistic binomial regressions between IADF frequency and tree-ring width for the three species (PIHA, ***Pinus halepensis***; PIPI, ***Pinus pinaster***; PIPN, ***Pinus pinea***).** IADF frequency and number of samples related to tree ring width for **(A)** *Pinus halepensis* (PIHA), **(B)** *Pinus pinaster* (PIPI), and **(C)** *Pinus pinea* (PIPN). TRW refers to tree-rings aligned from the narrowest to the widest ones.

The analysis of IADF frequency related to tree-ring widths showed a similar tendency of the three pine species with the occurrence of more IADFs in wide rings than in narrow or very large rings, especially in *P. halepensis* and *P. pinea*. Conversely, in *P. pinaster*, despite the decline showed in the largest tree rings, the frequency of IADFs was maintained above 40% in rings wider than 1 cm (**Figures 3A–C**). The highest IADF frequencies were observed for tree rings representing the percentiles 0.91, 0.89, and 0.80 of their ring widths for *P. halepensis*, *P. pinaster*, and *P. pinea*, respectively. The distributions of IADF frequency in relation to tree-ring width are bell shaped for all the three species. The comparison of IADF frequency and tree-ring width with sample depth showed that the highest values of IADF frequency are in the same range of ring widths (3–5 mm) for the three species regardless of the different growth rates, and it shows the increase in tree-ring width moving from the narrowest rings of *P. halepensis* to the widest ones of *P. pinaster* (**Figures 3A–C**).

### Geographical Pattern of IADF Frequency

**FIGURE 4:**
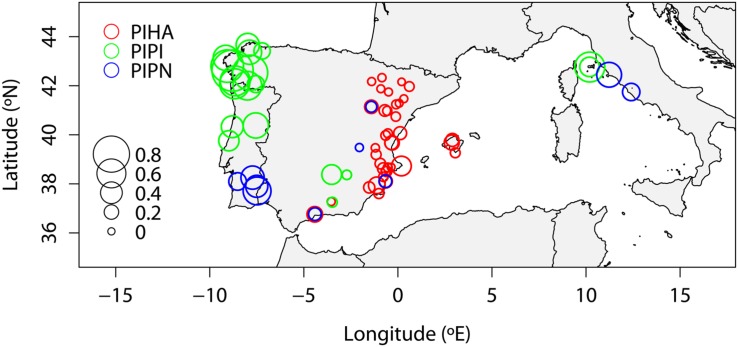
**Map of the mean IADF frequencies (IADF_f) of the sites of the network detrended by age (x axis: longitude –W, +E).** The amplitude of the circles is directly proportional to the frequency: wider circles are related to higher IADF frequency compared to smaller ones (PIHA, *Pinus halepensis*; PIPI, *Pinus pinaster*; PIPN, *Pinus pinea*).

The spatial distribution of mean IADF frequencies standardized by age showed a geographical pattern of fluctuations in the entire network (**Figure 4**). Data of raw and detrended frequency for each site are shown in the supporting material (Supplementary Table S2). *P. halepensis* in Spain was the species with the narrowest range of IADF frequencies, with a minimum of 0.3% and a maximum of 34.9% (**Figure 4**). *P. pinaster* showed the widest range of frequencies of IADFs ranging between 6.8 and 93.2%, with the highest values of frequency in north-west Spain (**Figure 4**). Finally, *P. pinea* IADF frequencies ranged between 2.2 and 53.6%, with the lowest values in Spain and maximum values in Portugal and in Italy (**Figure 4**).

### IADF Frequency and Climate

**FIGURE 5:**
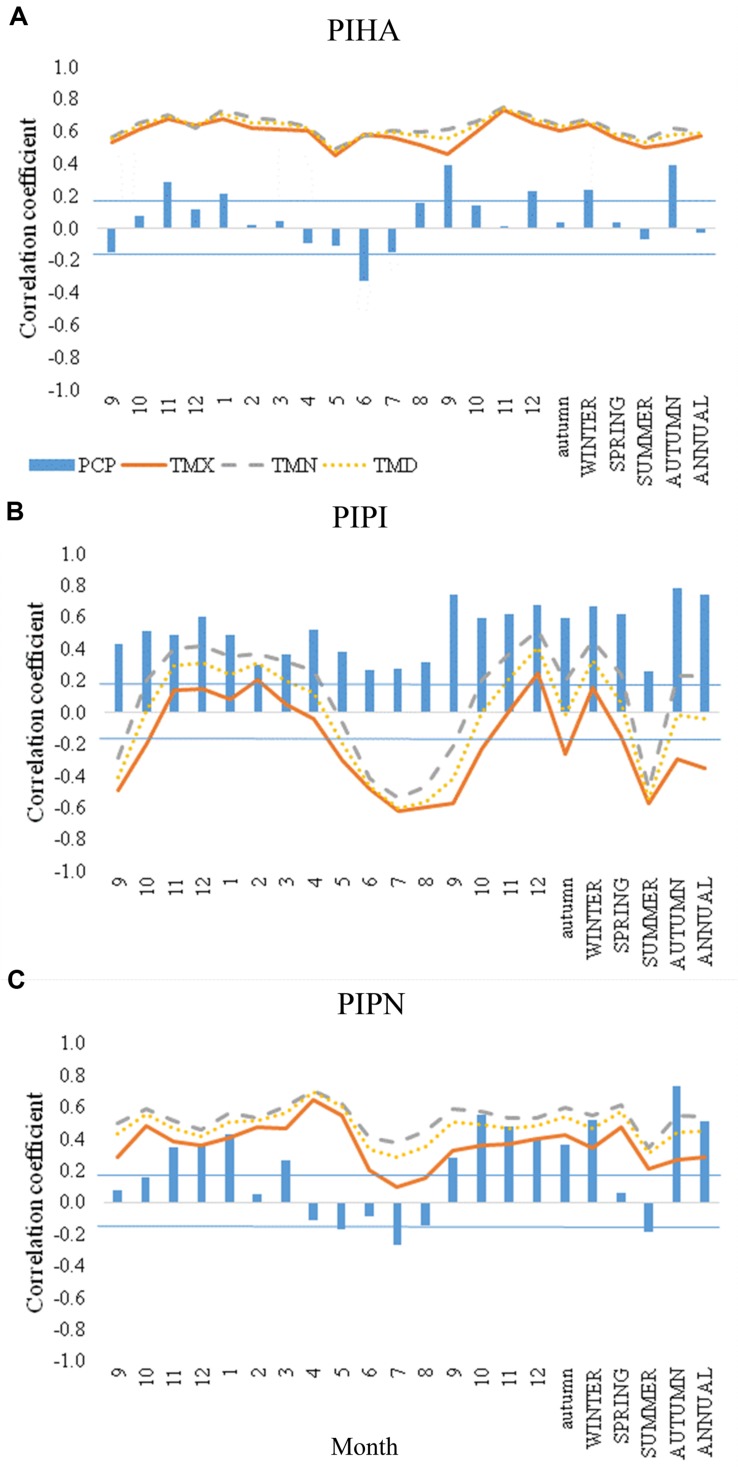
**Correlation coefficient values between IADF frequency, total precipitation (PCP) and maximum (TMX), minimum (TMN), and mean (TMD) monthly temperature for the three species [(A) ***Pinus halepensis*** (PIHA), (B) ***Pinus pinaster*** (PIPI), and (C) ***Pinus pinea*** (PIPN)].** IADF frequency is correlated with climate data at monthly and seasonal scale from September of previous year to December of the current year, and at annual scale. First numbers from 9 to 12 to the left of the graphs and lowercase letters refer to the previous year. Blue horizontal lines indicate the limits of the correlation coefficient (±0.196) for significance at *p* < 0.05 (*n* > 100).

In all the three species, autumn precipitation of the current growth year seemed to be the main climatic condition triggering IADF formation (**Figures 5A–C**). Correlation coefficients between precipitation in autumn and IADF frequency were 0.4 in *P. halepensis*, 0.8 in *P. pinaster*, and 0.7 in *P. pinea* (*p* < 0.05). Significant negative correlations with precipitation were found in June in *P. halepensis* (*r* = −0.3) and in July in *P. pinea* (*r* = −0.3), while *P. pinaster* IADF frequency was positively correlated with precipitation during the whole year (*p* < 0.05). IADF frequency was positively correlated with temperature throughout the year in *P. halepensis*, with values of 0.5–0.7 for T_min_, 0.5–0.7 for T_max_, and 0.5–0.7 for mean temperature. IADF frequency was also positively correlated with temperature throughout the year in *P. pinea*, with values ranging of 0.3–0.7 for T_min_, 0.1–0.7 for T_max_, and 0.3–0.7 for mean temperature. By contrast, a highly significant negative correlation with summer temperatures (from June to September) was observed in *P. pinaster* (*r* = −0.5 with T_min_, *r* = −0.6 with T_max_, and *r* = −0.6 with mean temperature), where the most negative correlations were found in July (*r* = −0.5 with T_min_, *r* = −0.6 with T_max_, and *r* = −0.6 with mean temperature; **Figure 5C**). Maximum autumn temperature also showed a negative correlation with IADF frequency in *P. pinaster* (*r* = −0.3), with a high negative correlation in September (*r* = −0.2 with T_min_, *r* = −0.6 with T_max_, and *r* = −0.4 with mean temperature).

## Discussion

### IADF Frequency – Tree-Ring Age Relationship

This study based on a large number of samples throughout the western Mediterranean Basin confirmed the presence of a strong relationship between IADF frequency and tree-ring age in all the analyzed pine species. An age trend toward a higher formation of IADFs in juvenile rings than in older ones was in agreement with previous studies showing that both tree-ring width and IADF frequency are age-dependent (Rigling et al., 2001, 2002; Bogino and Bravo, 2009; Hoffer and Tardif, 2009; Vieira et al., 2009; Olivar et al., 2012; Novak et al., 2013b; Campelo et al., 2015). A different timing and duration of xylem formation may explain the age-dependent IADF frequency. Indeed, the high frequency of IADFs in juvenile tree rings could be due to an earlier reactivation of the cambium and the consequent longer growing season, together with a fast physiological and morphological response to changing factors within the growing season (Villalba and Veblen, 1994; Vieira et al., 2009). On the other hand, high IADF frequency in young individuals could also be attributed to a higher sensitivity to environmental fluctuations: the shallower root systems of younger trees would favor IADF formation in response to changing water availability (Ehleringer and Dawson, 1992; Battipaglia et al., 2014). The relationship between a higher IADF frequency in tree rings and a shallower root system was also found by Pacheco et al. (2016), suggesting a higher sensitivity of the shallower rooted Spanish juniper to summer and autumn rains compared to the deeper rooted Aleppo pine in northeastern Spain. The strong relationship between IADF frequency and age highlights the necessity to overcome age trends in order to have an independent reconstruction of climate from IADFs (Novak et al., 2013b). In this paper, we show the importance of using a standardization method to obtain IADF series without the effect of the population structure and comparable among sites and species. Novak et al. (2013b) applied a standardization procedure to IADFs in *P. halepensis* to remove the effect of age, whereas Campelo et al. (2015) adopted a different method in *P. pinaster* to remove the effect of tree-ring width from IADF series. Here, a new approach was used to remove the effect of tree-ring age from IADF series across several species. This approach could be extended to other species across different geographical ranges and environments to facilitate the comparison of results and to gain univocal information.

### IADF Frequency Geographical Pattern and Tree-Ring Width Relationship

The map of mean detrended frequencies of IADFs of the studied sites helped to show the spatial distribution of frequency among species and geographical location, pointing out a potential climatic influence. The highest values of IADF frequency were found in the sites located at longitudes where Mediterranean climate is affected by oceanic influences, as Portugal and north-west Spain. The lowest values were found in the sites located in eastern Spain, where the climate ranges from Mediterranean to semiarid. High values of IADF frequency were also found for sites with warm Mediterranean climate as the ones located in Italy, southern Portugal and south-central Spain (Peel et al., 2007). The inter-specific analysis highlighted the relationship between IADF frequency and growth rate, and its relation with climate: *P. pinaster* was the species with the highest frequency of IADFs and the widest tree rings at the same time, growing in sites with the highest mean precipitations throughout the year and mild winter conditions. On the opposite, the lowest frequencies of IADFs were recorded in tree rings of *P. halepensis* that was also the species with the highest percentage of narrow rings, growing in sites characterized by the lowest values of mean precipitation throughout the year. In all the species studied, the relationship between IADF frequency and tree-ring width showed that IADFs tend to be more frequent in wide but not in the widest rings. This result is mainly in agreement with the recent finding of Carvalho et al. (2015) who suggests that the formation of IADFs in latewood of *P. pinaster* is predisposed by higher rates of cell production in spring which, in turn, increases the number of cells under enlargement after the summer drought, leading to the formation of wider rings. This could explain the case of *P. pinaster* in Portugal and north-west Spain, and *P. pinea* in Italy, under temperate and Mediterranean conditions, respectively, showing the highest IADF frequencies and the widest tree rings. Regarding the reason why IADF frequency decreases or remains stable in extreme wider tree rings, we hypothesize that extremely wider rings are usually formed during years with favorable conditions for tree growth throughout the growing season (Fritts, 1966) without fluctuations in environmental conditions, which is considered to be the main triggering factor of IADF formation (Carvalho et al., 2015). On the opposite, the low frequency of IADFs in narrow tree-rings may be attributed to particularly unfavorable conditions during the growing season (Fritts, 1966; Zubizarreta-Gerendiain et al., 2012). In this case, trees may not have enough reserves to allow the resumption of cambial activity as a response to favorable climatic conditions after the summer drought. This could explain the situation mainly found in *P. halepensis* and *P. pinea* sites in eastern Spain under a semi-arid Mediterranean climate (Peel et al., 2007), showing the lowest values of IADF frequency.

### Climatic Signal in IADF

The climate correlations allowed us to standardize the large amount of data from the three species growing under different microclimatic conditions and in populations with different structures, with the aim of analyzing common regional patterns over local ones. A common large-scale climatic factor driving IADF formation was autumn precipitation, with high values of correlation coefficients for all the analyzed species. These results agree with the hypothesis that the formation of IADFs in Mediterranean pines is mainly triggered by the resumption of cambial activity in response to the return of favorable conditions, such as autumn precipitation after summer drought (Campelo et al., 2007; de Luis et al., 2007, 2011a,b; Camarero et al., 2010; Novak et al., 2013a,b; Hetzer et al., 2014; Carvalho et al., 2015). A decrease in precipitation during summer, associated with an increase in temperatures, may influence cell division and expansion, slowing down cambial activity (Antonova and Stasova, 1997; Deslauriers and Morin, 2005; Vieira et al., 2014). Tracheids with narrow lumen and thick wall (latewood or latewood-like cells) are formed in response to low cell turgor during summer drought (Domec and Gartner, 2002). When water availability increases after autumn precipitation, differentiating cells can promptly re-acquire enough pressure (Wimmer et al., 2000; Abe et al., 2003; Rossi et al., 2009) for the enhancement of lumen enlargement (Wimmer et al., 2000), leading to earlywood-like cells (Carvalho et al., 2015; Vieira et al., 2015).

Late summer/autumn precipitation preceded by a drier period seemed to be the common triggering factor of IADFs in the three species. However, the species appear to be differently predisposed to the formation of IADFs depending on the geographic influence by peculiar climatic conditions experienced throughout the year.

Temperature seemed to play an important role for IADF formation in *P. halepensis* and *P. pinea* as confirmed by positive correlations between temperature and IADF frequency throughout the year. Favorable conditions of growth with high temperatures throughout the year could be a predisposing factor for the formation of IADFs in these two species, allowing them to efficiently react to seasonal fluctuations of precipitation. Moreover, temperatures suitable for growth throughout the year can induce a longer growing season, resulting in wider rings that are generally more prone to form IADFs (Deslauriers et al., 2008; Campelo et al., 2015). Regarding *P. pinaster*, living in the wetter sites of our network, precipitation seems to have a major role for IADF formation, as suggested by the positive correlations between precipitation and IADF frequency throughout the year. Furthermore, a mild dry summer was found to be a significant factor leading to the formation of IADFs in this species, as showed by the highly significant negative correlations of IADF frequency with summer-early autumn temperature. Favorable conditions for growth with wet conditions at the beginning of the growing season could facilitate *P. pinaster* to have a second period of cambial activity during autumn, after the summer drought, as suggested by Pacheco et al. (2016) for Spanish juniper. On the contrary, severe drought periods may prevent the formation of IADFs leading *P. pinaster* to an earlier stop of the growing season, unable to resume cambial activity in response to increased water availability (Vieira et al., 2014).

The methodological approach used in this study helped to link IADF frequency to climate conditions on a regional scale, but does not solve the question of the species-specific nature of their formation. There is evidence of a geographical/environmental gradient for *P. halepensis*, with more frequent IADFs in coastal than in inland or high elevated sites (Novak et al., 2013b). In our analysis, this trend was evidenced in *P. halepensis* but not in the other two pine species. The expansion of the network, especially by addition of new inland sites, could be useful to gain further insight on species-specific microclimate associations in IADF formation.

However, distribution ranges of the three species do not completely overlap, with wide geographical areas where the species do not coexist, making the comparison between species growing under the same weather conditions difficult. Thus, further research is needed using complementary approaches (including modeling) in order to disentangle the species-specific nature of IADF formation from the dependence on climate at a regional level.

Furthermore, several studies have shown the importance of the position of IADFs within the tree rings since it could reflect different climatic triggering factors of IADF formation and could also influence their frequency (Campelo et al., 2007; Battipaglia et al., 2010; De Micco et al., 2012). In the present work, we did not evaluate the relative position of IADFs within rings, since their identification is not straightforward as different methodological approaches exist (Campelo et al., 2007; De Micco et al., 2012, 2014). However, this kind of information is valuable to better understand the temporal link between IADFs and environmental factors, thus further improvements are needed to include this information in this type of analysis on a broad geographical scale.

## Conclusion

To date, a few studies have analyzed climatic influences on IADF formation across environmental gradients, and, to our knowledge, there is no study that combines spatial and temporal variations of IADF frequency and climate across a wide geographical range. Our results showed that tree-ring age has to be taken into account when analyzing IADFs-climate relationships because it plays an important role in IADF formation in Mediterranean pines.

A common interval of tree-ring width presenting the highest frequency of IADFs was found for the three species, with a more frequent formation of IADFs in tree rings moderately wide. Moreover, a common large-scale climatic factor driving IADF formation was found in autumn precipitation, demonstrating the potential of IADFs for climatic reconstructions. IADF formation was found to be lower in species living under Mediterranean to semi-arid conditions, where the frequency of narrow rings is higher (e.g., *P. halepensis* in eastern Spain). However, the highest frequency of IADF formation was found in species living in temperate sites with oceanic influence, where wetter conditions throughout the year associated with a moderately dry period in summer lead to the formation of wider tree rings (e.g., *P. pinaster* in Portugal and in north-west Spain). A proxy record of the intra-annual plastic response of wood traits on a large scale could add insights on global change studies as highlighted for anatomical parameters by Fonti et al. (2010). IADFs could be analyzed in a large tree-ring network and used as efficient indicators to predict the plastic adjustment of tree species to changing environmental conditions, especially in the climate hotspot of the Mediterranean ecosystems. Their occurrence in several Mediterranean species, particularly conifers as *Pinus* spp. enabled this pioneering study of IADFs on a wide network which might be further expanded to the entire Mediterranean Basin.

## Author Contributions

EZ, ML, and GB gave a substantial contribution to the conception and design of the study. All authors contributed to the supply of data for the network. ML and EZ were in charge for statistical analyses. EZ, VM, FC, and GB contributed to data analysis. EZ, ML, FC, VM, and GB gave substantial contribution to the interpretation of data. EZ wrote the main part of the manuscript. ML, FC, VM, GB, and EZ performed the critical revision of the work. All authors contributed to manuscript revision, read and approved the submitted version.

## Conflict of Interest Statement

The authors declare that the research was conducted in the absence of any commercial or financial relationships that could be construed as a potential conflict of interest.
