# Supplementary material for: Climatic Signals from Intra-annual Density Fluctuation Frequency in Mediterranean Pines at a Regional Scale
Source: Front Plant Sci. 2016 May 2;7:579. doi: 10.3389/fpls.2016.00579 (PMC4852653; doi:10.3389/fpls.2016.00579)
Supplement: Supplementary file 1 [file Table_1.DOC]

**Tab. S1** Details of each analyzed site of the network. Site numbers related to IADF chronologies newly processed in this study are marked with an asterisk.

|  | **Site number** | **Site** | **Site code** | **Country** | **Altitude** | **Longitude** | **Latitude** | **N of trees** | **N of samples** | **Starting year** | **End year** |
| --- | --- | --- | --- | --- | --- | --- | --- | --- | --- | --- | --- |
| ***Pinus halepensis*** |  |  |  |  |  |  |  |  |  |  |  |
|  | 1 | Alcoy (Penaguila) | ALC | Spain | 674 | -0.36 | 38.68 | 15 | 30 | 1852 | 2000 |
|  | 2 | Alloza | ALL | Spain | 595 | -0.57 | 40.98 | 15 | 31 | 1888 | 2006 |
|  | 3 | Ayerbe | AYE | Spain | 924 | -0.84 | 42.32 | 17 | 33 | 1946 | 2006 |
|  | 4 | Biar | BIA | Spain | 806 | -0.77 | 38.62 | 15 | 29 | 1927 | 2000 |
|  | 5 | Alcubierre | CAP | Spain | 738 | -0.50 | 41.75 | 22 | 36 | 1800 | 2006 |
|  | 6 | Caspe | CAS | Spain | 166 | 0.07 | 41.29 | 15 | 28 | 1845 | 2006 |
|  | 7 | Cartagena | CAT | Spain | 116 | -1.01 | 37.61 | 15 | 27 | 1915 | 2007 |
|  | 8 | Chiprana | CHI | Spain | 160 | -0.09 | 41.24 | 10 | 10 | 1900 | 2003 |
|  | 9 | Crevillente | CRE | Spain | 285 | -0.77 | 38.29 | 12 | 43 | 1832 | 2000 |
|  | 10 | Daroca | DAR | Spain | 937 | -1.41 | 41.14 | 14 | 28 | 1934 | 2006 |
|  | 11 | Ejea-Bardenas | EBA | Spain | 365 | -1.40 | 42.17 | 9 | 9 | 1909 | 2003 |
|  | 12 | Estopinian del Castillo | EST | Spain | 502 | 0.61 | 41.97 | 14 | 27 | 1964 | 2006 |
|  | 13 | Font de la Figuera | FHI | Spain | 680 | -0.93 | 38.83 | 14 | 28 | 1946 | 2011 |
|  | 14 | Alcoy (Font Roja) | FNT | Spain | 1022 | -0.54 | 38.67 | 14 | 24 | 1864 | 2005 |
|  | 15 | Fraga | FRA | Spain | 340 | 0.32 | 41.47 | 14 | 29 | 1844 | 2006 |
|  | 16 | Fuensanta | FUE | Spain | 138 | -1.12 | 37.94 | 14 | 26 | 1902 | 2007 |
|  | 17 | Gilet (Urbanizacion La Pinada) | GI2 | Spain | 140 | -0.31 | 39.67 | 14 | 26 | 1899 | 2011 |
|  | 18 | Gilet (SantEspirit) | GIL | Spain | 175 | -0.35 | 39.67 | 14 | 26 | 1892 | 2006 |
|  | 19 | El Grado | GRA | Spain | 168 | 0.20 | 42.16 | 15 | 30 | 1946 | 2006 |
|  | 20 | Guardamar | GUA | Spain | 15 | -0.65 | 38.10 | 11 | 39 | 1912 | 2000 |
|  | 21 | Jalance | JAL | Spain | 571 | -1.15 | 39.19 | 22 | 49 | 1863 | 2004 |
|  | 22 | Javea | JAV | Spain | 96 | 0.19 | 38.73 | 15 | 60 | 1924 | 2000 |
|  | 23 | MaigmoNorte | MAN | Spain | 845 | -0.64 | 38.52 | 35 | 70 | 1867 | 2009 |
|  | 24 | MaigmoSur | MAS | Spain | 762 | -0.60 | 38.50 | 23 | 39 | 1901 | 2009 |
|  | 25* | Montes de malaga | MDM | Spain | 135 | -4.42 | 36.76 | 13 | 26 | 1956 | 2012 |
|  | 26 | Montanejos | MON | Spain | 569 | -0.54 | 40.06 | 16 | 31 | 1955 | 2001 |
|  | 27 | Oliete | OLI | Spain | 530 | -0.69 | 40.99 | 15 | 28 | 1960 | 2006 |
|  | 28 | Oropesa | ORO | Spain | 1 | 0.12 | 40.06 | 15 | 30 | 1921 | 2003 |
|  | 29 | Mallorca (Binissalem) | PA1 | Spain | 120 | 2.86 | 39.69 | 13 | 26 | 1914 | 2011 |
|  | 30 | Mallorca (Caimari) | PA2 | Spain | 386 | 2.89 | 39.79 | 13 | 26 | 1888 | 2011 |
|  | 31 | Mallorca (Cap Salines) | PA3 | Spain | 14 | 3.05 | 39.27 | 12 | 23 | 1890 | 2011 |
|  | 32* | Cazorla - Puerta de Segura | PSC | Spain | 1030 | -2.72 | 38.36 | 15 | 28 | 1944 | 2012 |
|  | 33 | Puerto de Ragudo | RAG | Spain | 959 | -0.65 | 39.97 | 15 | 29 | 1964 | 2011 |
|  | 34 | Requena | REQ | Spain | 721 | -1.20 | 39.47 | 15 | 31 | 1789 | 2003 |
|  | 35* | Sierra de Huetor | SDH | Spain | 1120 | -3.54 | 37.27 | 15 | 30 | 1965 | 2012 |
|  | 36 | Sierra Espuña | SES | Spain | 846 | -1.52 | 37.86 | 16 | 29 | 1894 | 2007 |
|  | 37 | Villanueva de Gallego | VLL | Spain | 452 | -0.91 | 41.88 | 15 | 29 | 1878 | 2006 |
|  | 38 | Zorita | ZOR | Spain | 857 | -0.11 | 40.74 | 15 | 30 | 1832 | 2001 |
| ***Pinus pinea*** |  |  |  |  |  |  |  |  |  |  |  |
|  | 1 | Alcácer do Sal | ASPI | Portugal | 160 | -8.52 | 38.10 | 25 | 50 | 1896 | 2003 |
|  | 2 | Mértola | BEME | Portugal | 190 | -7.49 | 37.71 | 7 | 14 | 1917 | 2002 |
|  | 3 | Serpa | BESE | Portugal | 210 | -7.50 | 37.90 | 5 | 10 | 1929 | 2002 |
|  | 4* | Castelporziano | CP | Italy | 10 | 12.40 | 41.74 | 21 | 21 | 1897 | 2003 |
|  | 5 | Daroca | DAR | Spain | 937 | -1.41 | 41.14 | 15 | 31 | 1894 | 2007 |
|  | 6* | Duna Feniglia | DF | Italy | 10 | 11.22 | 42.44 | 14 | 14 | 1924 | 2003 |
|  | 7 | Portel | EVPO | Portugal | 270 | -7.73 | 38.25 | 5 | 10 | 1939 | 2002 |
|  | 8 | Guardamar | GUA | Spain | 15 | -0.65 | 38.10 | 14 | 27 | 1914 | 2003 |
|  | 9* | Montes de Malaga | MDM | Spain | 135 | -4.42 | 36.76 | 9 | 17 | 1888 | 2012 |
|  | 10* | Rubielos Altos | RBA | Spain | 800 | -2.05 | 39.48 | 12 | 23 | 1930 | 2012 |
| ***Pinus pinaster*** |  |  |  |  |  |  |  |  |  |  |  |
|  | 1 | Monte Aloia | ALO | Spain | 530 | -8.68 | 42.08 | 21 | 21 | 1967 | 2006 |
|  | 2 | A Barrela | BAR | Spain | 620 | -7.85 | 42.53 | 23 | 23 | 1967 | 2006 |
|  | 3 | A Capelada | CAP | Spain | 340 | -7.98 | 43.67 | 22 | 22 | 1967 | 2006 |
|  | 4 | Isla de Cortegada | COR | Spain | 20 | 8.78 | 42.62 | 22 | 22 | 1967 | 2004 |
|  | 5* | Despeñaperros | DES | Spain | 600 | -3.51 | 38.37 | 10 | 20 | 1964 | 2012 |
|  | 6 | Monte Insua | INS | Spain | 10 | -9.15 | 43.13 | 14 | 14 | 1967 | 2006 |
|  | 7 | Leiria | LEMG | Portugal | 33 | -8.99 | 39.76 | 40 | 80 | 1818 | 2006 |
|  | 8 | Marco da Curra | MCU | Spain | 590 | -7.88 | 43.37 | 19 | 19 | 1967 | 2006 |
|  | 9 | Muros | MUR | Spain | 155 | -9.07 | 42.80 | 21 | 21 | 1967 | 2006 |
|  | 10* | Isola d’Elba | PPFM | Italy | 420 | 10.20 | 42.77 | 8 | 8 | 1964 | 2007 |
|  | 11* | Isola d’Elba | PPTP | Italy | 460 | 10.18 | 42.77 | 9 | 9 | 1962 | 2007 |
|  | 12* | Cazorla – Puerta de Segura | PSC | Spain | 1050 | -2.72 | 38.36 | 10 | 20 | 1870 | 2012 |
|  | 13* | Sierra de Huetor | SDH | Spain | 1300 | -3.49 | 37.25 | 11 | 21 | 1942 | 2012 |
|  | 14* | Serra de Estrela | SEPP | Portugal | 1050 | -7.55 | 40.38 | 26 | 56 | 1907 | 2010 |
|  | 15 | Geres | SG | Portugal | 1500 | -8.00 | 42.00 | 18 | 36 | 1907 | 2007 |
|  | 16 | Tocha | TCHA | Portugal | 16 | -8.80 | 40.33 | 60 | 120 | 1953 | 2008 |
|  | 17 | Trabada | TRA | Spain | 640 | -7.22 | 43.42 | 23 | 23 | 1967 | 2006 |
|  | 18 | Verin | VER | Spain | 855 | -7.55 | 42.07 | 23 | 23 | 1967 | 2006 |
|  | 19 | Vigo | VIG | Spain | 365 | -8.65 | 42.20 | 19 | 19 | 1967 | 2006 |
