# Supplementary material for: Climatic Signals from Intra-annual Density Fluctuation Frequency in Mediterranean Pines at a Regional Scale
Source: Front Plant Sci. 2016 May 2;7:579. doi: 10.3389/fpls.2016.00579 (PMC4852653; doi:10.3389/fpls.2016.00579)
Supplement: Supplementary file 2 [file Table_2.DOCX]

**Tab. S2** Number of rings, number of IADFs, mean raw and age-detrended frequency of the sites of the network.

|  | **Site number** | **Site code** | **N of rings analyzed** | **N of rings with IADFs** | **Raw frequency (%)** | **Age-detrended frequency** |
| --- | --- | --- | --- | --- | --- | --- |
| ***Pinus halepensis*** |  |  |  |  |  |  |
|  | 1 | ALC | 3760 | 32 | 6.23 | 0.07 |
|  | 2 | ALL | 2878 | 110 | 2.32 | 0.25 |
|  | 3 | AYE | 1377 | 32 | 0.33 | 0.11 |
|  | 4 | BIA | 1771 | 207 | 3.82 | 0.58 |
|  | 5 | CAP | 2371 | 37 | 17.15 | 0.07 |
|  | 6 | CAS | 3514 | 33 | 12.20 | 0.07 |
|  | 7 | CAT | 2039 | 161 | 4.48 | 0.39 |
|  | 8 | CHI | 940 | 36 | 1.56 | 0.22 |
|  | 9 | CRE | 3456 | 213 | 0.94 | 0.34 |
|  | 10 | DAR | 991 | 170 | 3.83 | 0.84 |
|  | 11 | EBA | 571 | 12 | 2.10 | 0.10 |
|  | 12 | EST | 1044 | 65 | 1.73 | 0.30 |
|  | 13 | FHI | 1498 | 135 | 1.54 | 0.44 |
|  | 14 | FNT | 1936 | 78 | 4.74 | 0.22 |
|  | 15 | FRA | 3702 | 64 | 5.92 | 0.12 |
|  | 16 | FUE | 2094 | 539 | 0.85 | 1.39 |
|  | 17 | GI2 | 1737 | 321 | 11.69 | 1.01 |
|  | 18 | GIL | 2235 | 362 | 9.01 | 0.89 |
|  | 19 | GRA | 1505 | 5 | 4.03 | 0.02 |
|  | 20 | GUA | 2377 | 383 | 6.59 | 0.87 |
|  | 21 | JAL | 4308 | 284 | 5.01 | 0.39 |
|  | 22 | JAV | 3433 | 1197 | 8.43 | 1.69 |
|  | 23 | MAN | 4932 | 247 | 2.33 | 0.29 |
|  | 24 | MAS | 2692 | 227 | 6.16 | 0.43 |
|  | 25 | MDM | 1303 | 295 | 18.48 | 1.09 |
|  | 26 | MON | 1223 | 58 | 16.20 | 0.22 |
|  | 27 | OLI | 1049 | 128 | 16.11 | 0.57 |
|  | 28 | ORO | 2022 | 332 | 34.87 | 0.81 |
|  | 29 | PA1 | 2187 | 427 | 16.42 | 1.10 |
|  | 30 | PA2 | 2031 | 336 | 7.90 | 0.85 |
|  | 31 | PA3 | 2336 | 155 | 25.74 | 0.40 |
|  | 32 | PSC | 1644 | 92 | 7.98 | 0.27 |
|  | 33 | RAG | 1266 | 75 | 5.60 | 0.29 |
|  | 34 | REQ | 3858 | 90 | 22.64 | 0.30 |
|  | 35 | SDH | 1180 | 20 | 1.69 | 0.09 |
|  | 36 | SES | 2670 | 213 | 19.52 | 0.54 |
|  | 37 | VLL | 2597 | 40 | 16.54 | 0.08 |
|  | 38 | ZOR | 2725 | 122 | 6.64 | 0.29 |
| ***Pinus pinea*** |  |  |  |  |  |  |
|  | 1 | ASPI | 2854 | 771 | 27.01 | 1.57 |
|  | 2 | BEME | 799 | 428 | 53.57 | 2.77 |
|  | 3 | BESE | 578 | 221 | 38.24 | 1.96 |
|  | 4 | CP | 2174 | 623 | 28.66 | 1.78 |
|  | 5 | DAR | 2962 | 308 | 10.40 | 0.62 |
|  | 6 | DF | 956 | 483 | 50.52 | 2.61 |
|  | 7 | EVPO | 533 | 217 | 40.71 | 1.99 |
|  | 8 | GUA | 2114 | 319 | 15.09 | 0.88 |
|  | 9 | MDM | 1814 | 250 | 13.78 | 0.94 |
|  | 10 | RBA | 1346 | 29 | 2.15 | 0.12 |
| ***Pinus pinaster*** |  |  |  |  |  |  |
|  | 1 | ALO | 663 | 413 | 62.29 | 3.05 |
|  | 2 | BAR | 884 | 680 | 76.92 | 3.67 |
|  | 3 | CAP | 852 | 375 | 44.01 | 2.13 |
|  | 4 | COR | 825 | 769 | 93.21 | 4.50 |
|  | 5 | DES | 875 | 302 | 34.51 | 1.61 |
|  | 6 | INS | 484 | 240 | 49.59 | 2.38 |
|  | 7 | LEMG | 5213 | 1790 | 34.34 | 2.45 |
|  | 8 | MCU | 760 | 355 | 46.71 | 2.25 |
|  | 9 | MUR | 839 | 579 | 69.01 | 3.38 |
|  | 10 | PPFM | 327 | 206 | 63.00 | 2.97 |
|  | 11 | PPTP | 338 | 112 | 33.14 | 1.64 |
|  | 12 | PSC | 1970 | 133 | 6.75 | 0.34 |
|  | 13 | SDH | 1341 | 94 | 7.01 | 0.38 |
|  | 14 | SEPP | 4711 | 2292 | 48.65 | 2.92 |
|  | 15 | SG | 2756 | 1616 | 58.64 | 3.39 |
|  | 16 | TCHA | 5634 | 2283 | 40.52 | 1.92 |
|  | 17 | TRA | 915 | 228 | 24.92 | 1.19 |
|  | 18 | VER | 885 | 237 | 26.78 | 1.28 |
|  | 19 | VIG | 742 | 461 | 62.13 | 2.99 |
